# Diffuse Cutaneous Mucinosis in Dermatomyositis: A Case Report and Review of the Literature

**DOI:** 10.1155/2014/938414

**Published:** 2014-11-18

**Authors:** Alexandra Caitlin Perel-Winkler, Chris T. Derk

**Affiliations:** ^1^St. Luke's-Roosevelt Hospital Center, New York, NY 10025, USA; ^2^Division of Rheumatology, University of Pennsylvania, One Convention Boulevard, 8th Floor Penn Tower, Philadelphia, PA 19104, USA

## Abstract

We present the case of a patient with dermatomyositis and diffuse cutaneous mucinosis and give an up-to-date detailed review of all the published cases in the English literature describing the demographics, clinical picture, pathology management, and outcomes of this unique group of patients.

## 1. Introduction

Mucin (hyaluronic acid complex) is a protein normally found as part of the dermal connective tissues and it is produced by mast cells and fibroblasts. As hyaluronic acid holds water, in disease states where mucin production is increased, the dermal connective tissue becomes swollen and is described as myxedematous. It is not uncommon to have findings of microscopic cutaneous mucinosis in the setting of collagen vascular diseases and mucin deposition in the correct clinical setting can be considered as histologic evidence of dermatomyositis (DM) [1]. Clinically evident forms of mucinosis have been described in hypothyroidism, thyrotoxicosis, scleromyxedema associated with monoclonal gammopathies, scleredema related to diabetes, and lichen myxedematosus. Cases of secondary cutaneous mucinosis have been described in systemic lupus erythematosus, systemic sclerosis, and dermatomyositis, albeit infrequently [2–8]. We present a case of dermatomyositis with evidence of diffuse cutaneous mucinosis in a patient recently treated for nonsmall cell lung cancer (NSCLC) without evidence of recurrence.

## 2. Case

**Figure 1:**
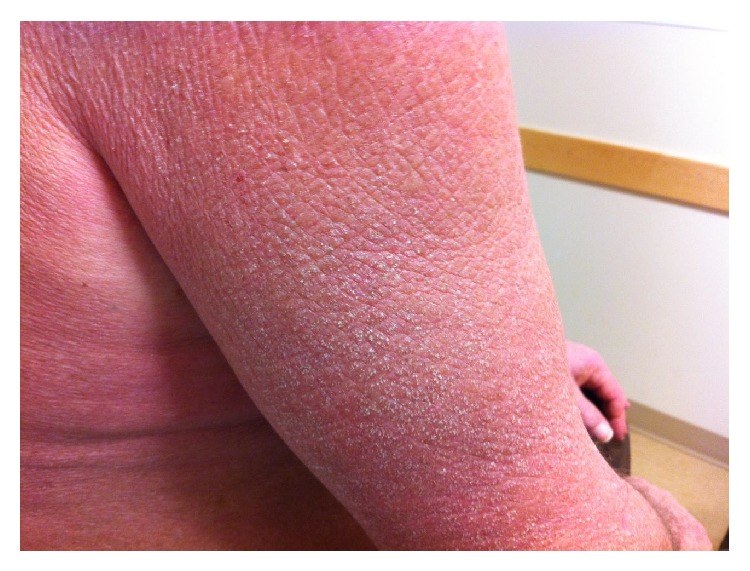
Cutaneous mucinosis: violaceous, scaly, and erythematous rash of the right arm.

**Figure 2:**
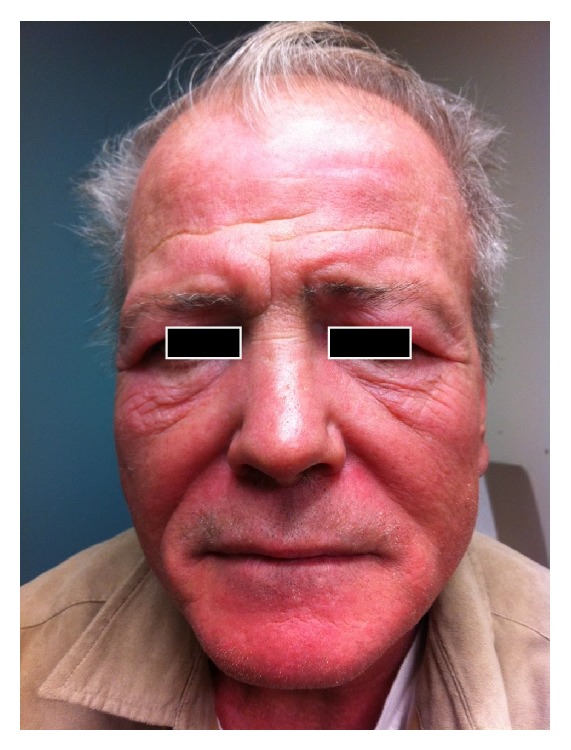
Cutaneous mucinosis: diffuse erythematous, violaceous rash of the face.

**Figure 3:**
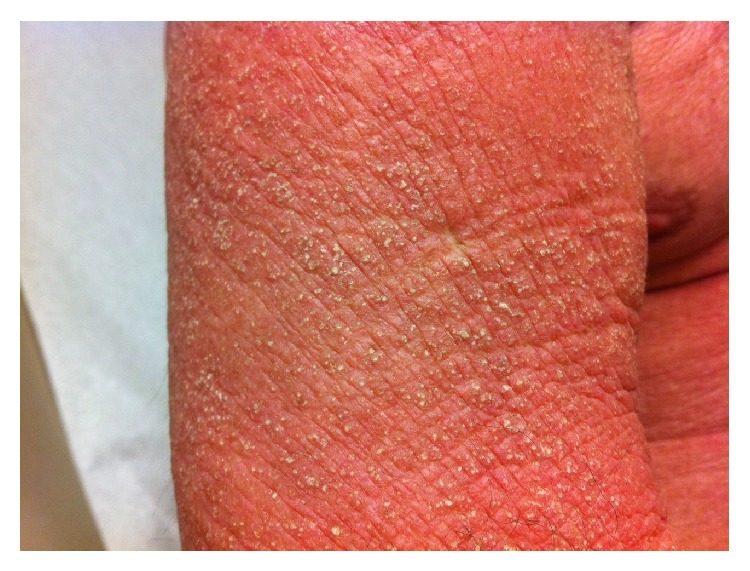
Cutaneous mucinosis: diffuse, scaly, and erythematous rash with white macules.

**Figure 4:**
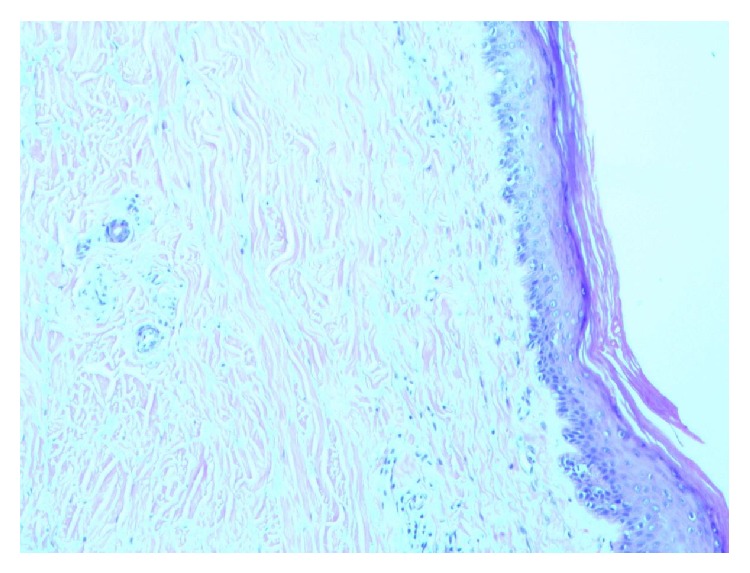
Skin biopsy: colloidal iron with hyaluronidase ×100. Dermal mucin deposition without fibroblast proliferation, with interface vacuolar changes.

**Figure 5:**
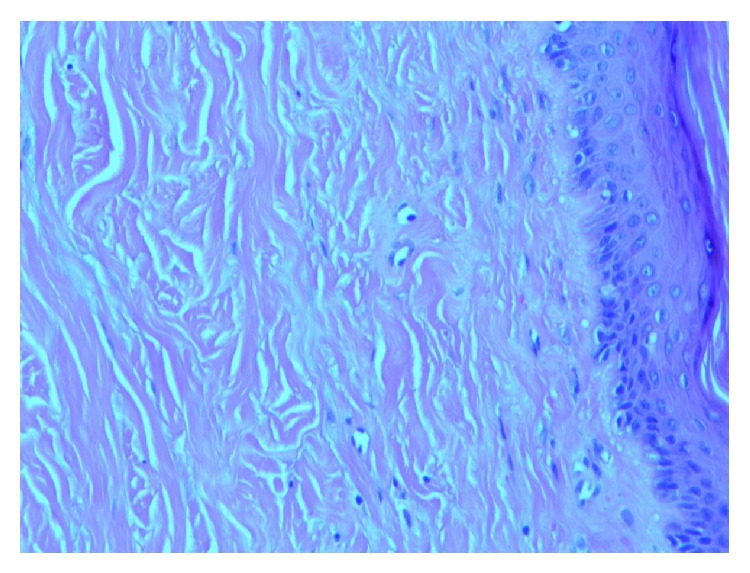
Skin biopsy: colloidal iron ×200: dermal mucin depositions without fibroblast proliferation.

A 57-year-old man with chronic obstructive lung disease, hypothyroidism, gastroesophageal reflux disease, and a prior history of NSCLC developed a pruritic, confluent, violaceous rash after cancer treatment. The patient was diagnosed with NSCLC in 2011 and was treated with paclitaxel and carboplatin and adjunctive radiation, with a restaging PET/CT scan showing excellent response. Four months after the completion of chemotherapy and radiation therapy the patient presented complaining of a pruritic rash. The rash first appeared on his hands and was noted to be consistent with Gottron's papules. Over the next nine months the rash worsened, and the patient developed violaceous erythema on his upper chest and back. Erythematous patches with white macules then developed on his lower legs, thighs, and buttocks. Three years after the treatment of his cancer, the patient had a diffuse, scaly, and erythematous rash on his arms (Figure 1), legs, buttocks, abdomen, neck, and face (Figure 2) with evidence of white macules (Figure 3) most prominent on the upper and lower extremities. Initial concern was for recurrence of his cancer; however, full body PET-CT revealed no new or active cancer. Skin biopsies showed evidence of interface dermatitis with sections of hyperkeratosis, mild spongiosis, interface vacuolar change, and dermal mucinosis without involvement of the panniculus or fascia (Figures 4 and 5). Muscle enzyme tests showed a normal creatinine phosphokinase level but an elevated aldolase at 9.5 U/L. A later full thickness biopsy performed showed evidence of interface dermatitis with mucin deposition. Two muscle biopsies were performed and HLA1 staining showed diffuse labeling of the sampled myofibers. Only one necrotic myofiber was isolated; otherwise the specimens were largely normal without diffuse myofiber necrosis, inflammation, or definite vacuolation. An MRI of the patient's femurs showed hyperenhancement in the obturator internus and externus muscles bilaterally and the proximal hamstrings (right greater than left), indicating some degree of inflammation. Immunoserologic results included a positive ANA of 1 : 640 with a speckled pattern and a positive Smith antibody (Ab). Of the myositis autoantibody panel, anti-Ku and anti-U1RNP were found to be positive. Other labs included a normal TSH and a slightly elevated gamma-globulin fraction of 1.7 g/dL (reference range 0.7–1.2 g/dL) with a normal immunofixation.

Dermatomyositis with cutaneous mucinosis was diagnosed in light of the physical exam findings, MRI evidence of inflammation, evidence of interface dermatitis, and mucin deposition on the skin biopsies and positive serologies. The demonstration of mucinosis without fibroblastic proliferation or dermal thickening supported a diagnosis of cutaneous mucinosis as opposed to scleromyxedema or systemic sclerosis.

Prior to presentation at our clinic, 3 years after the initial symptoms began, the patient had tried multiple medical treatments. He was initially treated with 5 mg of oral prednisone, which was quickly increased to 20 mg without success. Methotrexate was initiated at 7.5 mg weekly and then titrated to 15 mg weekly without response. Plaquenil 200 mg was tried for 2 months but the patient discontinued the treatment as he felt it had no effect. Once we diagnosed the patient with dermatomyositis and diffuse cutaneous mucinosis, we initiated 60 mg of prednisone per day which was tapered to 40 mg daily two weeks later due to side effects. Intravenous Immunoglobulin was initiated at 20 grams for 3 consecutive days every 6 weeks. At 3-month follow-up, the patient reported significant improvement in the amount of erythema and induration especially in the upper extremities and a decrease in the white macular lesions.

## 3. Discussion

### 3.1. Dermatomyositis and Cutaneous Mucinosis

Dermatomyositis is an inflammatory myopathy, which affects striated muscle and has cutaneous features. Typically a heliotropic rash, Gottron's papules, shawl sign, and erythematous plaques are some of the dermatologic manifestations; however, atypical cutaneous features, including plaque like mucinosis, have also been described [1]. The pathophysiology of dermatomyositis includes the expression of autoantibodies which target protein synthesis or translational particles in the muscle cell which triggers a humoral immune response. Activation of proinflammatory cytokines and chemokines leads to the migration of lymphoid cells to the perimysial and endomysial spaces; complement activation leads to the formation and deposition of membranolytic attack complexes onto endomysial capillaries. The result is microangiopathy and necrosis of endothelial cells leading to perivascular inflammation, muscle ischemia, and muscle fiber destruction [8, 13].

Mucin is a mucopolysaccharide produced by fibroblasts and consists of hyaluronic acid and sulfated glycosaminoglycans. Cutaneous mucinosis is subdivided into primary and secondary types; in primary, mucin deposition is the primary histologic feature and secondary, where mucin deposition is an additional finding to a primary clinicopathologic setting. Cutaneous manifestations of mucin can be focal or diffuse and are described as dermal or epidermal (follicular) [9, 14]. The pathophysiology of increased mucin deposition in connective tissue diseases is not completely understood and it is a rare finding. It is postulated that substances circulating in the serum, such as immunoglobulins, autoantibodies, or cytokines, stimulate glycosaminoglycan synthesis by fibroblasts leading to the production of mucin and its deposition in the skin [8, 12]. Pandya et al. linked the increased level of serum autoantibody titres with an increase in mucin lesions in patients with SLE [11, 15]. Interleukin-1 and interleukin-6 have also been shown to be elevated in patients with increased dermal mucin production in SLE and DM; however this is nonspecific as interleukins may be raised without evidence of mucinosis [2].

The concept of a hypoxic state contributing to the increased production of mucin has yet to be considered as part of the pathogenesis in DM. In cases of cutaneous mucinosis reported in the setting of venous insufficiency it has been hypothesized that reduced oxygen tension triggers chondrocytes to increase production of hyaluronic acid [16–18]. With perivascular inflammatory infiltrate, capillary obliteration, and myofiber necrosis as known sequelae of DM pathogenesis, it is conceivable that the biologic milieu of DM is hypoxic, and this may be a contributing factor towards mucin production.

Including our patient, there is a total of 12 cases in the English literature describing macroscopically evident cutaneous mucinosis in the setting of dermatomyositis (Table 1). Of these, three cases were associated with malignancy, and one patient had a history of autoimmune thyroiditis, inactive at the time of presentation.

Overall, clinical cutaneous manifestations of mucinous rashes are diverse: Chen, Requena, and Kaufmann describe plaque like skin changes, whereas Wang describes the rash as violaceous; Del Pozo and Johnson describe a distinctly papular rash. Most papers reported classic cutaneous findings of DM alongside the mucinous findings, with Gottron's papules and a heliotrope rash being common. Our patient had the most diffuse mucinous rash of the cases reported, involving the face, chest, back, and all extremities.

In the majority of cases, cutaneous symptoms preceded or occurred simultaneous to muscle weakness. Del Pozo et al. describe mucinous skin changes occurring four years after presentation and treatment of DM, and this is one of two cases where the mucinous skin changes did not resolve [2, 3]. In general, cutaneous lesions of mucinosis in the setting of DM seem to respond well to treatment when they appear in the early stages of disease. The majority of patients improved with oral steroids ± azathioprine, with resistant cases improving with IVIG [1]. Only one case did not describe improvement in cutaneous mucinosis despite lack of evidence of malignancy; in this case the mucinosis developed after DM had been successfully treated and did not respond to first line treatment [2]. One case was fatal due to respiratory complications of DM and recurrent infection due to long-term high dose steroid use [10]. Of note, in the latter two cases IVIG was not utilized per case documentation.

### 3.2. Dermatomyositis and Malignancy

DM has a clear temporal link with malignancy. Cancer may present in 15–30% of the adults with DM prior to or at diagnosis or during follow up. DM is most commonly associated with ovarian, breast, lung and colon cancer, melanoma, and non-Hodgkins lymphoma, with adenocarcinomas accounting for 70% of all associated tumors [19]. The pathophysiology relating DM and malignancy is unproven, but the leading proposed hypothesis is that of an autoimmune paraneoplastic mechanism. Myositis specific antigens (MSA), such as antisynthetase and antisignal recognition particle, have been shown to be expressed at low levels by normal muscles cells and are over expressed during regeneration of muscle fibers during DM [20]. A tumor may overexpress oncoproteins or antigens similar to the myositis antigens, which subsequently stimulate the immune system leading to a lymphocytic reaction causing autoantibody deposition and damage to myofibers [21–23]. Casiola-Rosen showed that solid tumors such as breast and lung may express exact MSA antigens. The damage to muscles causes a release of antigens from the muscle fibers themselves further sensitizing the immune system to the striated muscle. This theory is complemented by the previous theories discussed, correlating serologic antibody titers with DM activity.

As noted previously, malignancy was associated with DM and cutaneous mucinosis in 3 of the 12 cases in the English literature. This proportion of cases with cutaneous mucinosis related to malignancy is proportionate to the number of DM cases relating to malignancy reported in the literature generally (30%). It is unlikely that the cutaneous mucinosis is independently related to malignancy or that the presence of malignancy increases the chance of cutaneous mucinosis expressed in DM.

## 4. Conclusion

To date there are 12 cases describing macroscopically evident cutaneous mucin in DM. Our case describes a middle aged man with NSCLC in remission presenting with a puritic, diffuse, and violaceous rash. Histologic evidence showed mucin deposition in the dermis without dermal thickening alongside clinical, immunoserologic, and MRI findings consistent with DM, and even though our patient also had serologies suggestive of systemic lupus erythematosus, the predominant clinical picture was that of DM. The patient's cutaneous findings were highly resistant to first and second line treatments and only improved with the initiation of IVIG. While mucin deposition is a common microscopic finding in connective tissue diseases, it is rarely seen macroscopically. The pathophysiologic mechanism of cutaneous mucin production in these clinical scenarios is unclear. The link between hypoxic states and mucin deposition is a new concept, which has not been explored in the setting of dermatomyositis. Of the cases of cutaneous mucinosis and DM in the literature, the majority of cases improved with first line treatment for DM and in resistant cases positive results were seen using IVIG.

## Figures and Tables

**Table 1:** Literature review of all cases of cutaneous mucinosis in the setting of dermatomyositis.

Author, year, diagnosis	Demographics	Clinical description	Pathology (skin biopsy)	Associated condition(s)	Therapy and course
Johnson et al., 1973 [8] DM with lichen myxedematosus	35 African American males	Pruritic papular rash on upper and lower extremities, face, chest, and back. With perifollicular papules on forearms, hands, back, face and lower extremities, dysphagia and proximal muscle weakness of shoulders and pelvic girdle, and nail fold telangiectasia	Fragmented collagen with fibroblast proliferation and mucinous changes extending from the epidermis into the dermis with perivascular lymphocytosis and occasional histiocytes	None	80 mg oral prednisone daily with good response

Igarashi et al., 1985 [9] DM with cutaneous mucinosis	67 Japanese males	Poikilodermatous lesions on face, chest, and extremities	Skin bx: frayed and fragmented collagen bundles with mucinous material in between bundles when stained with alcian blue	Gastric cancer	Unknown

Requena et al., 1990 [3] DM with mucinosis	66 females	Erythematous, indurated plaque on hypogastric region of abdomen with irregular borders, heliotrope rash, violaceous rash on face, and Gottron's papules.	Amorphous mucinous material in epidermis and dermis which stained with alcian blue. The mucinous deposition caused thickening of the dermis and separation of collagen fibers	None	80 mg oral prednisone and 120 mg oral azathioprine daily for 4 weeks with slow taper improved malaise and weakness however only partial improvement of the abdominal mucinous plaque

Kaufmann et al., 1998 [1] Plaque like mucinosis	Case 1: 65 females	Case 1: erythematous plaques on extensor surface of upper extremities and left hip, muscle weakness of shoulder, and pelvic girdle	Case 1: separation of collagen bundles with perivascular lymphoplasmacytic infiltrate with abundant mucin deposition in the papillary and reticular dermis on staining with alcian blue	Case 1: none	Case 1: 80 mg oral prednisone daily, and 100 mg azathioprine daily with clinical response within 3 weeks, full resolution at 1 year
	Case 2: 37 females	Case 2: erythematous plaques over lateral surface of thighs bilaterally, weakness in shoulder and pelvic girdle, and clinical picture developed after fever arthralgia and malaise	Case 2: skin biopsy-separation of collagen vascular bundles with superficial and deep perivascular lymphoplasmacytic infiltrate of dermis with mucin between separated collagen bundles on Alcian blue staining	Case 2: obesity, history of autoimmune thyroiditis, preceded by viral prodrome. No malignancy detected	Case 2: 2 days of IVIG and high dose prednisone and then azathioprine, with excellent response
Del Pozo et al., 2001 [2] DM with cutaneous mucinosis	Case 1: 53 females	Case 1: small violaceous papules on upper extremities and chest; proximal muscle weakness	Case 1: skin biopsy showed hyperkeratosis, colloid bodies, and edema at the dermoepidermal junction with moderate perivascular lymphocytic infiltrate. Collagen bundles separated by mucin deposition	Case 1: ovarian adenocarcinoma	Case 1: recurrence despite prednisone, hydroxychloroquine, methotrexate, and azathioprine. Ovarian carcinoma treated with surgery, paclitaxel and cisplatin, no comment on status of DM or cutaneous findings after treatment
	Case 2: 44 females	Case 2: flesh coloured papules across flexural creases of palms and fingers	Case 2: skin biopsy-epidermal atrophy with perivascular lymphocytic infiltrate and moderate mucin deposition between collagen fibers	Case 2: none	Case 2: mucinosis developed after treatment for DM with 30 mg prednisone po and 250 mg hydroxychloroquine daily, lesions did not improve with this treatment

Tan et al., 2003 [7] DM with cutaneous mucinosis	65 Chinese males	Nontender erythematous plaques on neck, upper back and extensor surfaces of forearms bilaterally. Proximal muscle weakness developed 3 months after initial presentation	Mucin deposition between collagen bundles with surrounding superficial perivascular lymphocytic infiltrate without epidermal changes	Nasopharyngeal carcinoma	Skin lesions resolved after 2 months of radiation therapy

Chen et al., 2005 [10] Dermatomyositis with mucinosis and intestinal vasculopathy	21 Taiwanese females	Erythematous indurated mass on lower abdomen, labia majora and inner thigh with malar rash and periungual telangiectasia, heliotrope rash, Gottron's papules, proximal muscle weakness, and dysphagia	Atrophic epidermis with vacuolar alteration of basal keratinocytes, interstitial mucin deposition, and perivascular lymphocytic infiltrate of dermis and subcutaneous tissue	None	Prednisolone 60 mg/d IV with resolution of CPK however developed dysphagia and pulse steroid therapy initiated. Patient suffered complications and ultimately died after a long hospital course

Edward et al., 2007 [11] Amyopathic DM with mucinosis	31-year-old female	Puritic rash on flexor surface of forearms and chest, violaceous rash of face, Gottron's papules, and nail fold telangiectasia	Dermal accumulation of mucin without inflammatory infiltrate; no evidence of inflammatory myopathy	None	Improved with oral steroids

Wang, 2011 [12] DM with lichen myxedematosus	60-year-old male	Violaceous erythema on face, neck, and chest with flesh coloured papules on arms; muscle weakness of shoulders and dysphagia	Separation of collagen bundles with lymphocytic infiltrate of dermis and mucin deposition between collagen bundles demonstrated with alcian blue stain	None	Oral prednisone 40 mg daily with good response
